# Association of the combination of obstructive sleep apnea risk and sleep duration with ideal cardiovascular health metrics in patients undergoing hemodialysis

**DOI:** 10.1186/s12882-024-03517-x

**Published:** 2024-03-01

**Authors:** Hui Zhang, Ziwei Zhang, Yinjiao Zhao, Peiyu Song, Xiaoyu Chen, Peipei Han, Wei Ding, Liming Zhang, Chen Yu, Huizhi Ma, Qi Guo

**Affiliations:** 1 Jiangwan Hospital of Shanghai Hongkou District, Shanghai University of Medicine and Health Science Affiliated Rehabilitation Hospital, 1878 Sichuan North Road, 200081 Shanghai, China; 2 Department of Ultrasonography, Fujian Provincial Hospital, Fuzhou, China; 3 College of Rehabilitation Sciences, Shanghai University of Medicine and Health Sciences, Shanghai, China; 4 Department of Nephrology, Shanghai Ninth People’s Hospital, Shanghai Jiao Tong University School of Medicine, Shanghai, China; 5 Department of Nephrology, Zhabei Central Hospital of JingAn District of Shanghai, Shanghai, China; 6 Department of Nephrology, Tongji Hospital, School of Medicine, Tongji University, Shanghai, China; 7 Department of Rehabilitation Medicine, School of Health, Fujian Medical University, 1 Xuefu North Road, 350122 Fuzhou, China

**Keywords:** Cardiovascular disease, Hemodialysis, Ideal cardiovascular health metrics, Sleep

## Abstract

**Background:**

The purpose of this study was to explore the separate and combined associations of obstructive sleep apnea (OSA) risk and sleep duration with ideal cardiovascular health metrics in hemodialysis (HD) patients.

**Methods:**

470 HD participants (average: 59.48 ± 12.89 y, 281 men) were included in this study. Sleep duration was measured as self-reported average sleep time during the previous month. The OSA risk was assessed using the STOP-BANG questionnaire. Participants were divided into three groups based on the number of ideal cardiovascular health (CVH) metrics: 0–2,3–4, and 5–7. Ordinal logistic regression was conducted to model the associations of CVH metrics with sleep duration, OSA risk, and their combined effects by adjusting for specific covariates.

**Results:**

After adjusting for covariates, short sleep duration (< 7 h) (OR = 0.53; 95% CI [ 0.30, 0.92]) and OSA risk (OR = 0.58; 95% CI [0.32, 0.83]) were negatively associated with better CVH (ideal vs. intermediate; intermediate vs. poor), respectively. For HD patients with both short sleep duration and OSA risk, the odds of ideal CVH metrics were reduced by 72% (odds ratio 0.28 [95% CI 0.13, 0.60]).

**Conclusions:**

Short sleep duration and OSA risk are separately and jointly associated with poor CVH in hemodialysis patients. Suitable interventions for sleep may minimize the risk of developing cardiovascular disease.

**Supplementary Information:**

The online version contains supplementary material available at 10.1186/s12882-024-03517-x.

## Background

Cardiovascular disease (CVD) is the leading cause of mortality in patients with end-stage renal disease on hemodialysis(HD), and the relative risk of death from CVD in HD patients is 20 times higher than in individuals with normal renal function [1]. The American Heart Association (AHA) has defined 7 ideal cardiovascular health (CVH) metrics, which include healthy lifestyle behaviors and ideal metabolic measures, to promote optimal cardiovascular health in the population [2, 3]. Indeed, a higher number of ideal CVH metrics has been linked to a lower incidence of cardiovascular outcomes, such as heart failure and stroke, as well as other non-cardiovascular outcomes [4].

Although the concept of primary prevention of CVD to promote CVH has been widely recognized, preventive measures have still been insufficiently implemented. HD Patients suffer greater cardiovascular risks due to Sleep complaints, such as obstructive sleep apnea (OSA) and abnormal sleep duration, which are prevalent among HD patients [5, 6]. OSA may exacerbate oxidative stress, and endothelial dysfunction, and stimulate the sympathetic system through recurrent drops in oxygen saturation and sleep disturbances. On the other hand, abnormal sleep duration is usually closely related to circadian rhythm misalignment, adverse emotions, and underlying health issues, all of which increase the risk of CVD events in HD patients [7–9]. Several population-based studies have reported that both high risk of OSA and abnormal sleep duration are associated with poor CVH [2, 3, 9]. However, these associations have not been explored in HD patients. The individual and combined effects of OSA and sleep duration on ideal CVH metrics in HD patients remain to be established.

Therefore, this study aimed to explore the separate and combined associations of OSA risk and sleep duration with ideal CVH metrics in HD patients. This study may provide a reference for the role of good sleep habits in better CVH and may accelerate the process of clinical and public health promotion of CVH, particularly in HD patients.

## Methods

### Design and participants

This was a multicenter study conducted at hemodialysis centers in 8 hospitals in Shanghai and Suzhou, China. The study protocol was approved by the ethics committees of all hospitals and was conducted in accordance with the Declaration of Helsinki. We recruited 880 patients over 18 years old who underwent hemodialysis at least twice a week. The dialysis pathway was arteriovenous fistula and central venous catheter, with a dialysis session lasting 180–240 min. All participants and/or their legal guardian(s) volunteered to take part in the study and signed informed consent forms. The exclusion criteria were: (1) diagnosed with coronary heart disease; (2) diagnosed with stroke; (3) missing the necessary information in the present study. Finally, 470 HD patients were included in the study.

### Clinical data collection

Through face-to-face interviews conducted by trained research staff, this study utilized standard questionnaires to gather participants’ demographic characteristics, lifestyle, physical activity, dietary habits, and emotional states. Height and weight were measured using a standard protocol. Body mass index (BMI) was calculated as weight/height^2^ (kg/m^2^). Sociodemographic variables include age, sex, education level, marital status, living status and job category. Education levels were categorized as follows: no formal education (0 year), primary school education (1-6 years), secondary school education (7-9 years), and high school or above (9 years). The job category is divided into manual work, non-manual work, and mixed work. Information on smoking (never smoked or quit smoking in the past 12 months, current smoker) and drinking (non-drinkers, drinkers) was also obtained from the questionnaire. Physical activity was assessed using the short form of the International Physical Activity Questionnaire (IPAQ-SF), and we have described the methods of IPAQ in detail in a previous study [10]. Depressive symptoms were assessed using the Patient Health Questionnaire-9 (PHQ-9) [11]. Diet categories were divided into light salt intake, moderate salt and heavy salt intake by asking their preference for salty or light flavors [12].

Body composition, like extracellular water (ECW), intracellular water, and total body water volume, was measured using a bioelectrical impedance analyzer (Inbody S10; BioSpace, Seoul, Korea). Overhydration (OH) was subsequently calculated by using the equations in the device [13]. Hemodialysis patients were stratified into normal hydration (OH/ECW < 7%), and fluid overload (OH/ECW ≥ 7%) based on this calculation [14].

A blood sample was obtained from the antecubital vein collected between 7 am and 9 am after an overnight fast. Blood samples were collected in potassium EDTA tubes (Vacationer; BD, Oxford, the United Kingdom). TCH (total cholesterol), TG (triglycerides), LDL (low-density lipoprotein cholesterol), and other routine blood biochemical indexes were analyzed in the same way as in our previous [10]. SpKt/V (Single-pool Kt/V), calculated using the Daugirdas formula: spKt/V = − ln ((Ct/C0) − 0.008×t) + (4–3.5× (Ct/C0)) ×ΔBW/BW, where C0 is BUN at the beginning of the HD session (mmol/L), Ct is BUN at the end of the session (mmol/L), t is treatment time (hours), ΔBW is pre-HD body weight minus post-HD body weight, and BW is post-HD body weight(kg). Finally, spKt/V was automatically calculated from the database.

### Assessment of sleep

Sleep duration was obtained through the fourth question of the Pittsburgh Sleep Quality Index (PSQI), which was assessed by trained research staff: “In the last week, how many hours do you sleep on average per day?” [15]. The response was a numerical variable, categorized into three groups for analysis: <7 h (short sleep), 7–9 h (optimal sleep), and > 9 h (long sleep) [11]. The risk of obstructive sleep apnea was assessed by using the STOP-BANG questionnaire (SBQ) [5, 16]. The STOP-BANG questionnaire is a valid and effective screening tool for OSA in different populations and demonstrates a high identification ability [16]. We classified individuals with STOP-BANG score of 3 or higher as being at risk for OSA [5]. In addition, sleep quality was inquired by reporting, “How would you describe your sleep quality in the last month?” (very well, good, not enough, or very poor). Participants were also asked to report whether they had taken sleep medication in the last month before going to bed (types of sleep medication have been described in a previous study [10].

### Assessment of ideal cardiovascular health metrics

We used the AHA definitions of ideal cardiovascular health metrics [3]. These metrics consist of 7 modifiable health behaviors and factors, which were divided into three levels (ideal = 2 points; intermediate = 1 point; poor = 0 points). CVH was assessed based on hemodialysis patients’ self-reported smoking status, dietary habits, physical activity, and medication for hypertension, diabetes, and hyperlipidemia, in conjunction with their blood indicators. In addition, since salt intake has a significant impact on the cardiovascular health of the Chinese population, the dietary habits in this study will primarily focus on salt intake rather than vegetable intake [17]. Briefly, the ideal health behaviors include: (1) ideal smoking status, which means never smoking or quitting smoking more than 12 months ago; (2) ideal BMI, which is BMI < 25.0 kg/m^2^; (3) ideal physical activity, which means moderate to vigorous physical activity ≥ 150 min/week; (4) ideal dietary habits, characterized by light salt intake. The ideal health factors include: (1) ideal Total Cholesterol (TCH), Untreated TCH < 5.18 mmol/L; (2) ideal Fasting Plasma Glucose (FPG), Untreated FPG < 5.6 mmol/L; (3) ideal Blood Pressure (BP), untreated Systolic Blood Pressure (SBP) < 120 mmHg/ Diastolic Blood Pressure (DBP) < 80 mmHg. We sum up the number of ideal cardiovascular health metrics for each patient, ranging from 0 to 7. Patients were categorized into poor (0–2), intermediate (3–4), and ideal (5-7) [3].

### Statistical analysis

Data were presented as mean ± SD and median (quartiles) for continuous variables, and frequency (percentages) for categorical variables. Analysis of variance and Kruskal-Wallis test corrected by Bonferroni were used for continuous variables, and χ2 test was used for categorical variables.

Ordinal logistic regressions were performed without considering the interactions, and CVH groups were then modeled according to sleep duration only and OSA risk only, adjusting for the covariates mentioned previously (models 1–3, respectively). In model 4, we further examined the the relationship between sleep duration and ideal cardiovascular health metrics stratified according to OSA risk. ORs were calculated using the function for linear combinations of coefficients, with the sleep duration of ‘7–9 h’ and OSA risk(-) being considered as the reference categories. All analyses were performed using IBM’s Statistical Package for the Social Sciences (SPSS), version 25. A *p*-value < 0.05 was considered statistically significant.

## Results

The basic characteristics of the 470 HD patients (281 men and 189 women; average age: 59.48 ± 12.89 years) included in this study are presented based on the number of Ideal Cardiovascular Health Metrics in Table 1. They were divided into three groups according to the number of ideal CVH metrics: 0–2 (poor CVH, *n* = 88), 3–4 (intermediate CVH, *n* = 273), and 5–7 (ideal CVH, *n* = 109, reference group). Compared with patients with ideal CVH, poor and intermediate CVH individuals had worse CVH indicators, including BMI, smoking, physical activity, salty diet, glucose, blood lipids and blood pressure. HD patients with poor and moderate CVH who had higher age, spkt/v and potassium, they were also more likely to be male, drinker, fluid overload, shorter sleepers and at a higher OSA risk.

**Table 1:** Characteristics of participants by number of Ideal Cardiovascular Health Metrics

Variables	Total (*n* = 470)	Poor (0–2) (*n* = 88)	Intermediate (3–4) (*n* = 273)	Ideal (5–7) (*n* = 109)ref	*P*-value
Age, y	59.48 ± 12.89	60.39 ± 12.58	60.33 ± 12.89*	56.61 ± 12.84	0.029
BMI, kg/m^2^	23.14 ± 3.53	25.31 ± 3.66*	22.96 ± 3.48*	21.85 ± 2.70	<0.001
Male, n(%)	281(59.8)	75(85.2)*	169(61.9)*	37(33.9)	<0.001
Educational level, n(%)					0.493
0 year	18(3.8)	1(1.1)	13(4.8)	4(3.7)	
1–6 years	81(17.3)	11(12.5)	49(18.0)	21(19.3)	
7–9 years	165(35.2)	37(42.0)	90(33.1)	38(34.9)	
>9 years	206(43.7)	39(44.3)	121(44.1)	46(42.2)	
Widowed, n(%)	34(7.2)	2(2.3)	23(4.9)	9(8.3)	0.132
Living alone, n(%)	28(6.0)	6(6.8)	14(5.1)	8(7.4)	0.652
Job category, n(%)					0.030
Manual work	137(29.1)	29(33.0)	77(28.2)	31(28.7)	
Non-manual work	192(40.9)	26(29.5)	110(40.3)	56(51.8)	
Mixed work	141(30.0)	33(37.5)	86(31.5)	22(20.4)	
Drinker, n(%)	56(11.9)	16(18.2)*	33(12.1)*	7(6.4)	0.040
Smoker, n(%)	97(20.6)	31(35.2)*	62(22.7)*	4(3.7)	<0.001
Fluid overload, n(%)	247(52.6)	50(56.8)*	128(27.2)*	68(62.4)	0.019
MIS,score	3.80 ± 2.40	3.62 ± 2.39	3.83 ± 2.43	3.86 ± 2.37	0.755
CCI, score	3.23 ± 1.34	3.42 ± 1.36	3.23 ± 1.41	3.08 ± 1.11	0.214
IPAQ, Met/wk	1386(693,3465)	693(396,1386)*	1386(693,3251)*	3238(1569, 5656)	<0.001
Sp Kt/v	1.40 ± 0.32	1.32 ± 0.38*	1.39 ± 0.31	1.47 ± 0.27	0.006
Blood biochemical indexes					
SBP,mmHg	152.74 ± 25.46	156.61 ± 21.77*	153.58 ± 25.58	147.58 ± 27.30	0.033
DBP, mmHg	84.23 ± 16.09	82.05 ± 16.94	85.09 ± 15.64	83.82 ± 16.44	0.292
TCH, mmol/L	3.97 ± 1.30	3.82 ± 1.05	4.05 ± 1.47	3.91 ± 0.98	0.305
TG, mmol/L	1.81 ± 1.49	2.93 ± 2.91	2.54 ± 8.08	1.81 ± 1.49	0.434
LDL, mmol/L	2.34 ± 0.80	2.23 ± 0.61	2.37 ± 0.86	2.34 ± 0.77	0.345
HDL, mmol/L	0.97 ± 0.28	0.85 ± 0.30*	0.98 ± 0.28	1.03 ± 0.26	<0.001
GLU, mmol/L	7.50 ± 3.55	9.54 ± 4.26*	7.52 ± 3.42*	5.78 ± 2.03	<0.001
Hemoglobin, g/L	111.70 ± 15.97	109.68 ± 15.89	112.63 ± 16.31	111.01 ± 15.10	0.282
Albumin, g/L	40.44 ± 14.03	39.39 ± 3.04	39.88 ± 3.23	42.72 ± 28.53	0.149
CRP, mg/L	6.18 ± 14.06	6.92 ± 12.57	5.82 ± 12.31	6.49 ± 18.87	0.816
BUN, mmol/L	25.87 ± 6.49	25.93 ± 7.57	25.72 ± 6.45	26.20 ± 5.65	0.805
Scr, µmoI/L	1012 ± 274	1069 ± 332	1004 ± 266	984 ± 233	0.074
UA, µmoI/L	450.25 ± 96.91	458.69 ± 106.02	449.51 ± 97.24	444.92 ± 88.15	0.648
Phosphate, mmol/L	1.97 ± 0.82	2.24 ± 0.22	2.58 ± 5.34	2.29 ± 0.24	0.415
Potassium, mmol/L	4.88 ± 0.83	4.62 ± 0.76	4.88 ± 0.84	5.07 ± 0.80	0.003
Total calcium, mmol/L	2.45 ± 4.07	2.24 ± 0.22	2.58 ± 5.34	2.29 ± 0.24	0.712
Sleep characteristic					
Sleep duration					0.004
<7 h	215(45.7)	56(63.6)*	115(42.1)	44(40.4)	
7-9 h	207(44.0)	25(28.4)*	126(46.2)	56(51.4)	
>9 h	48(10.2)	7(8.0)	32(11.7)	9(8.3)	
NOA	1.64 ± 1.36	1.65 ± 1.46	1.73 ± 1.37	1.40 ± 1.25	0.107
Sleep mediation, n(%)	111(23.6)	23(26.1)	66(24.2)	22(20.2)	0.586
Sleep quality, n(%)					0.056
Very well	142(30.2)	18(20.5)	90(33.0)	34(31.2)	
Good	188(40.0)	35(39.8)	105(38.5)	48(44.0)	
Not enough	64(13.6)	20(22.7)*	35(12.8)*	9(8.3)	
Very poor	76(16.2)	15(17.0)	43(15.8)	18(16.5)	
OSA risk, n(%)	234(49.8)	64(72.7)	135(49.5)	35(32.1)	<0.001

Data are mean ± SD, percentage or median (quartile 1, quartile 3)

*P*-value comparison across number of cardiovascular health metrics groups using analysis of variance or Kruskal-Wallis for continuous variables and χ2 test for qualitative variables

*p < 0.05, compared with subjects with ideal cardiovascular health metrics (5–7)

Abbreviations: BMI, body mass index; MIS, malnutrition inflammation score; CCI, charlson comorbidity index; IPAQ, international physical activity questionnaire; Sp Kt/v,single-pool Kt/V-urea; SBP,systolic blood pressure; DBP,diastolic blood pressure; TCH,total cholesterol; TG,triglycerides; LDL,low density lipoprotein cholesterol; HDL,high density lipoprotein cholesterol; GLU, glucose; CRP,C-reactive protein; BUN,blood urea nitrogen; Scr,serum creatinine;UA,uric acid;NOA,number of awakenings; OSA,obstructive sleep apnea

The ORs of ideal CVH metrics across different levels of sleep duration and OSA risk are shown in Table 2. In ordinal logistic regression models, for HD patients who slept < 7 h, the proportional odds of having better CVH (ideal vs. intermediate; intermediate vs. poor) were 47% lower (odds ratio 0.53 [95% CI 0.30, 0.92]; *P* = 0.025, Model 3), compared with 7-9 h sleep duration. Furthermore, for HD patients at risk for OSA, the proportional odds of having better CVH (ideal vs. intermediate; intermediate vs. poor) were 42% lower (odds ratio 0.58 [95% CI 0.32, 0.83]; *P* = 0.007, Model 3).

**Table 2:** Ordinal logistic regression analyses for the associations between number of Ideal Cardiovascular Health Metrics and Sleep characteristic

Variables	Model 1		Model 2		Model 3	
	OR(95%CI)	*P*	OR(95%CI)	*P*	OR(95%CI)	*P*
OSA						
OSA risk (+)	0.38(0.25,0.57)	<0.001	0.62(0.39,0.97)	0.035	0.58(0.32,0.83)	0.007
OSA risk (-)	Reference		Reference		Reference	
Sleep duration						
<7 h	0.55(0.36,0.85)	0.007	0.55(0.35,0.85)	0.007	0.53(0.30,0.92)	0.025
7-9 h	Reference		Reference		Reference	
>9 h	0.61(0.32,1.19)	0.145	0.54(0.33,1.26)	0.194	0.77(0.37,1.58)	0.474

Dependent variable: Ideal Cardiovascular Health Metrics(poor, intermediate, ideal)

Model 1: crude model

Model 2: adjusted for Age, Sex

Model 3: adjusted for Age, Sex, Job category,Ddrinker, IPAQ, Fluid overload, Spkt/v, HDLandPotassium

The association of the combination of sleep duration and OSA risk with ideal CVH metrics was included in Table 3. The sleep duration ‘7–9 h’ and OSA risk(-) were both viewed as reference categories. In general, short sleeper (< 7 h) combined OSA risk (+) was associated with lower ORs of better CVH among HD patients (odds ratio 0.28 [95% CI 0.13, 0.60]; *P* = 0.001).

**Table 3:** Ordinal logistic regression analyses for the associations between number of Ideal Cardiovascular Health Metrics and sleep duration stratified according to OSA risk

	Sleep duration					
	<7 h		7-9 h		>9 h	
	OR(95%CI)	*P*	OR(95%CI)	*P*	OR(95%CI)	*P*
OSA risk(+)	0.28(0.13,0.60)	0.001	0.58(0.29,1.16)	0.123	0.36(0.11,1.15)	0.084
OSA risk (-)	0.57(0.27,1.20)	0.141	Reference		0.89(0.35,2.25)	0.804

Dependent variable: Ideal Cardiovascular Health Metrics(poor, intermediate, ideal)

Covariate variable:Age,Sex, Job category, Drinker, IPAQ, Fluid overload, Spkt/v,HDL and Potassium

## Discussion

This study evaluated the relationship between sleep characteristics and ideal cardiovascular health (CVH) metrics in HD patients. After adjusting for age, sex, job category, drinker, IPAQ, fluid overload, spkt/v, HDL, and potassium, this study showed that short sleep duration(< 7 h) and OSA risk were independently associated with poor cardiovascular health metrics (0–2 components), in comparison to ideal cardiovascular health metrics (5–7 components). Furthermore, the assessment of the combined effects of sleep duration and OSA risk on ideal CVH metrics in our study indicated the presence of OSA risk discrepancies in the correlation between sleep duration and ideal CVH metrics in HD patients. To our knowledge, this is the first study to explore the separate and combined associations of sleep duration and OSA risk with ideal cardiovascular health metrics in HD patients.

Consistent with previous studies [4, 18], our study found that only 23.19% of HD patients met 5–7 ideal CVH metrics, less than half of the general Chinese population. In fact, traditional risk factors for cardiovascular disease, such as hypertension, diabetes, obesity, and lack of exercise, are more common in HD patients. Dialysis-related factors, such as anemia, inflammation, oxidative stress, and fluid overload, also contribute to an increased incidence of CVD events [1]. However, the results of our study showed that despite regular visits to hospitals, HD patients have a higher risk of cardiovascular disease than the general population, and their condition is not well-controlled. Cardiovascular disease is the most common complication and the leading cause of death in HD patients. Therefore, more attention should be focused on the prevention and control of CVD in HD patients.

Sleep complaints, such as OSA risk and short sleep duration, have been linked to an increased risk of CVD events in several epidemiological studies [3, 7–9]. Notably, the prevalence of OSA and short sleep duration increases with the deterioration of chronic kidney disease. The risk of OSA in HD patients may be 10 times higher than in the general population, and the proportion of short sleep duration in HD patients is also substantially higher than in the general population [6, 8, 10, 19]. The characteristic airflow limitation and repetitive arousal in OSA were related to periodic hypoxemia and re-oxygenation, as well as enhanced sympathetic activation. These factors may lead to oxidative stress, endothelial dysfunction, and an inflammatory response that ultimately damage cardiovascular health [7]. Similar to the findings of this study, the American Heart Association Go Red for Women Strategically Focused Research Network (AHA GRFW SFRN) population study study showed that short sleep duration (< 7 h) and OSA risk were associated with 60–289% higher odds of having poor CVH [9]. The mechanisms of short sleep duration associated with CVD events include the mutual changes in leptin and ghrelin circulation levels. These changes can increase appetite and reduce energy consumption, potentially promoting the occurrence of obesity [20]. Moreover, short sleep duration was associated with dialysis-related fluid overload, circadian rhythm disorders, inflammatory response activation, etc., thereby increasing the risk of CVD [8, 9].

In HD patients, the management of cardiovascular risk factors is crucial, particularly modifiable ones. The ideal cardiovascular health metrics defined by the American Heart Association (AHA) do not include sleep but encompass several traditional cardiovascular risk factors (blood pressure, TCH, total cholesterol, fasting plasma glucose and BMI) and non-traditional cardiovascular risk factors (smoking status, dietary habits, and physical activity). Beyond their potential influence on clinical cardiovascular risk factors, sleep complaints may also be associated with other modifiable lifestyle behaviors, including diet, physical activity and smoking. Previous studies have reported that short sleepers and OSA patients are more likely to have irregular eating habits, such as fewer main meals, frequent intake of energy-dense snacks, or consuming more animal viscera, fried foods, and salted foods [21, 22]. The influence of OSA and sleep duration on physical activity (PA) is not well-established. Only one study reported that OSA was linked to lower physical activity levels, while the quality of evidence for the impact of exercise on sleep intervention in patients with chronic kidney disease was low, indicting a potential behavioral mechanism for the cardiovascular impairments associated with sleep complaints [23, 24].

Data from the Henan Rural Cohort and NHANES also showed that people who slept less were more likely to smoke [2, 8]. Among OSA patients, the proportion of frequent and occasional smokers was significantly higher compared to non-OSA patients [22]. There is weak evidence that insomnia exacerbates smoking and discourages quitting. Genetic correlations and bidirectional causal effects between sleep complaints and smoking indicate that sleep could be a potential target for smoking treatment and prevention [25].

Considering the interactive and confounding effects, we further explored the associations between the combination of OSA risk and sleep duration in relation to ideal CVH metrics in HD patients. The results of this study showed that for HD patients with short sleep duration and OSA risk, the proportional odds of ideal CVH were reduced by 72%.The combination of OSA risk and short sleep duration appears to be more detrimental to cardiovascular health. Although recent studies have shown that OSA patients with short sleep duration are closely related to hypertension, central obesity, and insulin resistance, the relationship between OSA combined with short sleep and cardiovascular disease remains unclear [26, 27]. Tetyana et al. found that among 21% of subjects without OSA (Apnea Hypopnea Index < 5) but at risk for OSA, subjects with an objective sleep duration of 4.9 h had a lower risk of CVD events than those with an objective sleep duration of 6.4 h [28]. Nevertheless, the results of Xiaoman et al. were quite different. OSA showed a strong correlation with moderate-to-high 10-year CVD risk, however, there was no significant association between sleep duration and CVD risk in the population with OSA [29]. Since most previously published studies on OSA risk, sleep duration, and cardiovascular health were cross-sectional, we were unable to determine the role of OSA risk and sleep duration. In this study, we explored the basic characteristics of HD patients in different sleep duration groups according to OSA risk stratification. We observed that poor sleep characteristics tended to be concentrated, and HD patients with OSA risk combined with short sleep duration had the worst sleep (including the use of sleep medications and sleep quality)(see Supplementary Table 1). Poor CVH metrics may be related to the cumulative impact of multiple sleep problems. HD patients with multiple sleep problems should embrace a healthy lifestyle and consistently monitor cardiovascular indicators to effectively engage in primary prevention of CVD.

### Strength and limitations

This study is the first to demonstrate that in HD patients with OSA risk and short sleep duration are significantly associated with poor CVH, respectively. Moreover, the co-existence of OSA risk and short sleep has a greater impact on poor CVH. Our assessment of clinical and lifestyle risk factors for CVD is very rigorous. The validity and reliability of the questionnaire are verified by standardized and widely used questionnaires [11, 16]. Standardized anthropometric and blood pressure measurements are carried out by trained research staff and blood indicators are assessed using standardized protocols [10]. Furthermore, we thoroughly examined the relationship between OSA risk, sleep duration and ideal CVH metrics. The results of this study could imply the significant role of sleep in the primary prevention of CVD in HD patients, and offer guidance for clinical intervention.

This study also has some limitations. Firstly, information about OSA risk and sleep duration was obtained from self-reported questionnaires rather than objectively assessed sources. Polysomnography (PSG) is the gold standard for diagnosing obstructive sleep apnea (OSA), and can also accurately measure sleep duration. However, the detection process of standard PSG is complex and needs to be carried out in a professional sleep laboratory, which is difficult to promote in a large sample population [30]. The STOP-BANG questionnaire and self-reported sleep duration are the most commonly used methods for subjectively assessing sleep. The STOP-BANG questionnaire has been proven to be an effective tool for screening OSA risk in different medical populations. It also shows a certain degree of consistency with PSG measurement assessment and self-reported sleep duration [31, 32]. Secondly, the sample size of this study is moderate, which limits the ability to analyze ideal CVH metrics stratified by different sleep problems. Therefore, the investigation into the combined effects of different sleep problems was exploratory. Finally, our study is a cross-sectional study, indicating a significant correlation between OSA risk and sleep duration are significantly correlated with ideal CVH metrics. However, the lack of temporality limits the ability to infer causality, highlighting the need for more prospective studies with larger sample sizes.

## Conclusion

In conclusion, sleep may be a key factor associated with the maintenance of ideal cardiovascular health metrics. We observed that OSA risk and short sleep duration are associated with poor CVH in hemodialysis patients. It is worth noting that OSA combined with short sleep duration showed a greater detrimental effect on poor CVH than OSA alone. This study contributes to the growing body of evidence suggesting that sleep characteristics should be taken into account when evaluating the risk of cardiovascular disease in HD patients. It is plausible to consider that sleep interventions may play a role in the primary prevention of cardiovascular disease. Future studies should explore a causal relationship between OSA, sleep duration and ideal CVH metrics.

### Electronic supplementary material

Below is the link to the electronic supplementary material.

Supplementary Material 1

## Data Availability

All data and materials relevant to the study are included in the article.
