# Supplementary material for: Association of the combination of obstructive sleep apnea risk and sleep duration with ideal cardiovascular health metrics in patients undergoing hemodialysis
Source: BMC Nephrol. 2024 Mar 1;25:77. doi: 10.1186/s12882-024-03517-x (PMC10908049; doi:10.1186/s12882-024-03517-x)
Supplement: Supplementary file 1 — Supplementary Material 1 [file 12882_2024_3517_MOESM1_ESM.docx]

Supplementary table 1: Characteristics of hemodialysis grouped according to three sleep duration groups stratified by OSA risk

| Variables | OSA risk (－) | | | P-value | OSA risk (＋) | | | P-value |
| --- | --- | --- | --- | --- | --- | --- | --- | --- |
|  | ＜7h(n=98) | 7-9(n=108) | ＞9h(n=30) |  | ＜7h(n=117) | 7-9h(n=98) | ＞9h(n=19) |  |
| Age, y | 61.39±13.17* | 53.55±13.45 | 60.14±11.71 | ＜0.001 | 59.91±11.39 | 62.04±11.49 | 67.47±15.29 | 0.029 |
| BMI, kg/m^2^ | 22.31±3.62 | 22.17±3.28 | 22.78±3.31 | 0.689 | 24.10±3.40 | 24.04±3.60 | 22.46±3.13 | 0.152 |
| Male, n(%) | 55(56.1) | 65(60.1) | 16(53.3) | 0.689 | 86(73.5) | 80(81.6) | 13(68.4) | 0.258 |
| Educational level, n(%) |  |  |  | 0.302 |  |  | * | 0.006 |
| 0 years | 6(6.1) | 5(4.6) | 3(10.0) |  | 0(0) | 2(2.0) | 2(10.5) |  |
| 1-6 years | 13(13.2) | 20(18.5) | 9(30.0) |  | 16(13.7) | 16(16.3) | 6(31.6) |  |
| 7-9 years | 35(35.7) | 34(31.4) | 10(33.3) |  | 43(36.8) | 35(35.7) | 8(42.1) |  |
| ＞9 years | 44(44.8) | 49(45.3) | 8(26.6) |  | 58(49.6) | 44(44.8) | 3(15.8) |  |
| Widowed, n(%) | 11(11.2) | 6(5.5) | 3(10.0) | 0.333 | 8(6.8) | 5(5.1) | 1(5.3) | 0.851 |
| Living alone, n(%) | 5(5.1) | 5(4.6) | 1(3.3) | 0.921 | 10(8.5) | 3(3.1) | 1(5.3) | 0.238 |
| [Job](javascript:;) [category](javascript:;) |  |  |  | 0.004 |  |  |  | 0.451 |
| [manual](javascript:;) [work](javascript:;) | 41(41.8) | 27(25.0) | 3(10.0) |  | 39(33.3) | 22(22.4) | 5(26.3) |  |
| [non-manual](javascript:;) [work](javascript:;) | 32(32.6) | 47(43.5) | 20(66.6) |  | 43(36.7) | 41(41.8) | 9(47.4) |  |
| Mixed work | 25(25.5) | 34(31.4) | 7(23.3) |  | 35(29.9) | 35(35.7) | 5(26.3) |  |
| Drinker, n(%) | 8(8.1) | 13(12.0) | 1(3.3)* | 0.121 | 16(13.7) | 13(37.1) | 6(31.6) | 0.105 |
| Smoker, n(%) | 10(10.2) | 17(15.7) | 6(20.0) | 0.292 | 30(25.6) | 29(29.6) | 4(21.1) | 0.675 |
| Fluid overload, n(%) | 51(52.0) | 59(54.6) | 17(56.6) | 0.814 | 56(47.9) | 55(56.7) | 8(42.1) | 0.814 |
| MIS,score | 4.03±1.97 | 3.78±2.66 | 4.03±2.78 | 0.737 | 3.58±2.57 | 3.76±2.12 | 4.10±2.64 | 0.644 |
| CCI, score | 2.97±0.95 | 2.91±1.19 | 3.13±1.38 | 0.639 | 3.47±1.43 | 3.63±1.58 | 3.10±1.14 | 0.339 |
| IPAQ, Met/wk | 1386(693,3032) | 2226(813,4746) | 1386(528，3892) | 0.121 | 1386(693,3186) | 1746(693,4079) | 1386(693,1848)* | 0.006 |
| sp Kt/v | 1.42±0.29 | 1.46±0.27 | 1.38±0.28 | 0.378 | 1.39±0.44 | 1.32±0.24 | 1.41±0.221 | 0.262 |
| Blood biochemical indexes | | | | | | | | |
| SBP,mmHg | 156.56±25.11 | 149.67±26.41 | 146.34±28.37 | 0.078 | 157.50±23.91* | 149.07±24.49 | 153.26±27.56 | 0.044 |
| DBP, mmHg | 84.95±15.77 | 87.20±17.52 | 82.34±19.06 | 0.344 | 85.23±14.47 | 80.43±15.34 | 80.73±15.96 | 0.055 |
| TCH, mmol/L | 4.18±1.91 | 3.92±0.97 | 4.06±1.02 | 0.458 | 3.94±1.16 | 3.77±0.97 | 4.37±1.66 | 0.103 |
| TG, mmol/L | 2.12±1.29 | 2.05±1.61 | 2.52±1.24 | 0.808 | 3.26±12.34 | 2.20±1.41 | 2.78±4.50 | 0.684 |
| LDL, mmol/L | 2.40±0.71 | 2.29±0.71 | 2.30±0.87 | 0.510 | 2.42±0.86 | 2.21±0.78 | 2.53±1.20 | 0.141 |
| HDL, mmol/L | 1.04±0.26 | 0.95±0.26 | 0.93±0.26 | 0.038 | 0.96±0.29 | 0.92±0.28 | 1.02±0.45 | 0.359 |
| GLU, mmol/L | 7.01±3.41 | 6.71±2.62 | 7.48±3.13 | 0.454 | 8.39±4.04 | 7.50±3.48 | 9.14±5.16 | 0.120 |
| Hemoglobin, g/L | 110.32±14.13 | 111.66±15.76 | 116.84±13.72 | 0.119 | 112.01±19.26 | 110.36±14.69 | 114.93±12.65 | 0.520 |
| Albumin, g/L | 39.87±3.21 | 40.33±3.31 | 40.21±2.80 | 0.588 | 41.81±27.62 | 39.47±3.15 | 40.71±2.70 | 0.686 |
| CRP, mg/L | 5.51±16.92 | 4.01±5.22 | 3.94±3.75 | 0.676 | 7.63±15.91 | 7.52±16.50 | 9.23±14.58 | 0.925 |
| BUN, mmol/L | 25.66±6.45 | 26.03±6.32 | 25.00±5.59 | 0.725 | 26.37±6.55 | 26.40±5.93 | 21.35±9.85* | 0.007 |
| Scr, μmoI/L | 965.58±275.98 | 1024.42±234.29 | 1028.74±223.42 | 0.203 | 1029.97±292 | 1045.91±269.35 | 823.41±360.67* | 0.009 |
| UA, μmoI/L | 431.08±88.66* | 467.82±92.91 | 450.48±72.28 | 0.031 | 441.74±92.38 | 471.56±102.28 | 385.75±125.26* | 0.003 |
| phosphate, mmol/L | 1.93±0.59 | 1.87±0.61 | 2.04±0.62 | 0.394 | 2.06±0.67 | 1.99±0.56 | 1.870.69± | 0.421 |
| potassium, mmol/L | 4.93±0.91 | 4.72±0.76 | 4.76±0.72 | 0.229 | 4.98±0.83 | 4.96±0.76 | 4.87±1.23 | 0.881 |
| Total calcium, mmol/L | 2.24±0.23 | 3.12±8.51 | 2.23±0.23 | 0.509 | 2.29±0.25 | 2.24±0.26 | 2.10±0.19 | 0.013 |
| Ideal CVH metrics group |  |  |  | 0.101 |  |  |  | 0.022 |
| 0-2 | 14(14.2) | 8(7.4) | 1(3.3) |  | 42(35.9) | 16(16.3) | 6(31.6) |  |
| 3-4 | 59(60.2) | 60(55.5) | 22(73.3) |  | 57(48.7) | 67(68.4) | 11(57.9) |  |
| 5-7 | 25(25.5) | 40(37.0) | 7(23.3) |  | 18(15.4) | 15(15.3) | 2(10.5) |  |
| Other Sleep characteristic | | | | | | | | |
| NOA | 1.68±1.38 | 1.32±1.30 | 2.00±1.48 | 0.029 | 1.72±1.41 | 1.65±1.32 | 2.05±1.26 | 0.501 |
| Sleep mediation, n(%) | 29(29.5)* | 14(12.9) | 6(20.0) | 0.013 | 41(35.0)* | 17(17.3) | 3(15.8) | 0.007 |
| Sleep quality, n(%) | * |  |  | ＜0.001 | * |  |  | ＜0.001 |
| Very well | 13(13.2) | 48(44.4) | 17(56.7) |  | 19(16.2) | 39(39.7) | 6(31.6) |  |
| Good | 50(51.0) | 46(42.5) | 7(23.3) |  | 34(29.0) | 42(42.8) | 8(42.1) |  |
| Not enough | 20(20.4) | 9(8.3) | 1(3.3) |  | 23(19.6) | 9(9.1) | 3(15.8) |  |
| Very poor | 15(15.3) | 5(4.6) | 5(16.6) |  | 41(35.0) | 8(8.1) | 2(10.5) |  |

Data are mean±SD, percentage or median (quartile 1, quartile 3).

P-value comparison across number of cardiovascular health metrics groups using analysis of variance or Kruskal-Wallis for continuous variables and χ2 test for qualitative variables.

*p<0.05, compared with subjects with 7-9 h sleep duration

Abbreviations: BMI , body mass index ; MIS, malnutrition inflammation score; CCI, Charlson comorbidity index; IPAQ, international physical activity questionnaire; sp Kt/v, single-pool Kt/V-urea ; SBP, systolic blood pressure; DBP, diastolic blood pressure; TCH, total cholesterol; TG, triglycerides; LDL, low density lipoprotein cholesterol; HDL, high density lipoprotein cholesterol; GLU, glucose; CRP, C-reactive protein; BUN, blood urea nitrogen; Scr, serum creatinine ;UA, uric acid; NOA, number of awakenings; OSA, obstructive sleep apnea.
